# Prevalence and associated factors of active trachoma among 1–9 years old children in Deguatemben, Tigray, Ethiopia, 2018: community cross-sectional study

**DOI:** 10.1186/s12886-020-01394-0

**Published:** 2020-04-15

**Authors:** Gebremeskel Reda, Dejen Yemane, Aregawi Gebreyesus

**Affiliations:** 1 Tigray Regional Health Bureau, Mekelle, Tigray Ethiopia; 2 College of Health Sciences, Mekelle University, P.O.BOX:1871 Mekelle, Tigray Ethiopia

**Keywords:** Prevalence and associated factors, Active trachoma, Tigray, Ethiopia

## Abstract

**Background:**

Trachoma is a contagious infection of the eye. World Health Organization recommended three rounds of mass drug administration in districts where the prevalence of trachomatous follicular (TF) is ≥10% in children aged 1–9 years. Mass drug distribution was given to residents for three consecutive years with more than 90% coverage. However, the prevalence and associated factors of active trachoma in the study community after the intervention was not yet determined. Thus, this deals with the prevalence and associated factors of active trachoma among children aged 1–9 years.

**Methods:**

We conducted a Community based cross-sectional study among 502 children aged 1–9 in March 2018 in Deguatemben. A multi-stage sampling technique was applied. Selected children were examined for trachoma using 2.5x binocular loupe and graded based on the WHO simplified grading system. Mothers were interviewed for factors associated with trachoma using a structured questionnaire. Data was entered on Epi-Info and exported to SPSS for analysis. Both descriptive and inferential analyses were done with 95% confidence intervals (CIs) at a *p*-value < 0.05 for the final model.

**Results:**

The prevalence of active trachoma was found 21.5% (95% CI: 17.8–25.1%). Being 1 to 4 years old [AOR (95% CI) = 6.81(2.00–23.11)], not washing face [AOR (95% CI) =9.31(1.13–77.66)], not using soap [AOR (95% CI) =5.84(1.87–18.21)], unclean face [AOR(95% CI) = 18.22(4.93–69.32)] and mother’s knowledge [AOR (95% CI) =0.06(0.02–0.19)] were found as independent predictors.

**Conclusion:**

The prevalence declined from the baseline, but it is still a public health problem in the district. Personal-related factors were found to be associated with the disease. Health education of “Facial cleanness” and related factors is recommended to increase knowledge of the mothers on their children’s care in addition to the provision of antibiotics.

## Background

Trachoma is the leading cause of blindness resulted from repeated trachoma infection [1]. Infected eye secretions with an active infective stage of trachoma are the main source of trachoma infection and mode of transmission includes; direct eye to eye spread, contaminated fingers, indirect spread through sharing towels, pillows, and eye seeking flies [2].

According to World Health Organization (WHO) trachoma grading scheme trachoma is classified as Trachomatous inflammation follicular (TF**)**, Trachomatous inflammation intense (TI), Trachomatous scarring (TS), Trachomatous trichiasis (TT**)** and Corneal opacity (CO) [3, 4]. Active trachoma includes WHO grades of TF and/ or TI. Endemicity of trachoma is classified based on TF prevalence as non-endemic (< 5%), hypo-endemic (≥5 and < 10%), mesoendemic (≥10 and < 30%) and hyper-endemic (≥30%) [5].

WHO Global Elimination of Trachoma (GET2020) Alliance recommends the implementation of the surgery, antibiotics, facial cleanness and environmental improvements (SAFE) strategy which tackles the disease through surgery to correct trichiasis, antibiotics to treat chlamydial infection and facial cleanliness and environmental improvements to suppress transmission of infection to eliminate the disease by 2020 [3].

Globally, about 150 million people suffer from active trachoma and 6 million people are blind due to its devastating complications and it is endemic in 42 countries: in Africa, in the Middle East, Central and South America, Asia and Australia with 200 million populations at risk living in these endemic countries [6–8].

Ninety-three percent (170 million) of the worlds’ population at risk of trachoma live in Africa and Africa carries 72% of the global burden of trichiasis [9]. In Ethiopia 2.8 million people have low vision, 1.2 million lost their vision, 9 million children 1–9 years old have active trachoma and the prevalence of active trachoma is 40.1% [10].

All 34 rural woredas of Tigray had been mapped and revealed that 1.7 million (about 40%) people live in hyper-endemic districts of the region and active trachoma prevalence among 1–9 years old children is 26.1%. Deguatemben woreda is one of the 12 woredas with the prevalence of active trachoma among children 1–9 years old ≥30% [11].

Different risk factors have been identified for trachoma from different settings which include; poor personal hygiene, lack of sanitary facilities and lack of accessible water for washing [12–16].

The WHO recommends annual mass treatment (MDA) of entire communities with oral azithromycin for at least 3 years if the prevalence of TF in 1–9-year-olds within a district or community exceeds 10% and with coverage of at least 80% of the population [17]. After the completion of the global trachoma mapping project (GTMP) in Tigray, Light for the World (the NGO that works to eliminate trachoma in Ethiopia) began supporting the regional trachoma elimination program in 2014 using the SAFE strategy and all trachoma endemic districts of the region are covered by the surgery and antibiotics (S & A) components of SAFE strategy. The prevalence of active trachoma among 1–9 years old children was > 30% and all residents of the woreda are taking Azithromycin mass drug administration starting in 2014 and it takes three rounds and will continue to five rounds. The MDA coverage of the woreda was 96% in the 1st round 2014, 95% in the 2nd round 2015, and 93% in the 3rd round 2016 [7].

The prevalence and associated factors of active trachoma in the study area are not yet determined and the WHO recommends the assessment of active trachoma among 1–9 years old children after MDA of antibiotics. Thus, this study will contribute to the regional and district efforts of trachoma prevention and control through assessment of the level of prevalence in the study area, the implementation of MDA, and identifying factors associated with active trachoma in the community. The findings of this study will also help for the planning of prevention, control and elimination efforts of the disease. This study was aimed to assess the prevalence of active trachoma and associated factors among 1–9 years old children 1 year after three rounds of azithromycin mass administration in Deguatemben woreda.

## Methods

### Study area and period

The study was conducted in Deguatemben woreda, one of the 4 woredas in the Southeast zone of Tigray Regional state that has 24 kebeles (smallest administrative unit in Ethiopia) in which one of them is urban. The current population size of the woreda is estimated to be 150,254 based on the 2007 population census. The woreda has one primary hospital, 5 health centers, and 24 health posts according to the woreda data. There is no ophthalmic professional in any of the health facilities. The study was conducted from 1st March to 30th March 2018.

### Study design and population

We conducted a community-based cross-sectional study among 1–9 years old children. All 1–9 years old children in the selected kebeles were our source of population and we include children from the age of 1–9 years old who were lived for at least 6 months in the study area. Children who are unable to undergo physical examination due to medical illness were excluded from the study.

### Sample size and sample size determination

The sample size was determined for both objectives. For the first objective, we were determined using a single population proportion formula n = (Zα/2)^2^p(1 − p)/d^2^, where n is the sample size, z is the standard normal deviation, set at 1.96 (for 95% confidence interval [CI]), d is the desired degree of marginal error (taken as 0.05) and p is the estimated prevalence of active trachoma (18%) taken from similar community [15]. The minimum sample size is 227 and expecting a 10% nonresponse rate and a design effect of two the final sample size is 500. We also determined the second objective, using the associated factor of active trachoma from a study done on the previous time. Sampling was determined based on the double proportion formula on the software of Epi Info StatCalc version 7 after considering the following assumptions; 95% confidence interval (CI), 80% power, 1:1 ratio of exposed to non-exposed group, odds ratio (OR) of 2.2 and taking the proportion-1 of 55.2% and proportion-2 of 32.8% number of preschool children as a factor for active trachoma [13]. Finally, with an expected non-response rate of 10% and the design effect (2.0) the maximum sample size was 502. Then we took the largest sample size that is 502.

### Sampling techniques and procedure

The study employed multi-stage with a stratified sampling technique. Initially, the stratification of kebeles into rural and urban strata was made. Seven kebeles (1 urban and 6 rural) were selected from the total kebeles in the district using a simple random sampling technique. Then, probability proportional to size (PPS) used to allocate children aged 1–9 years from each kebeles. Finally, children aged 1–9 years old were selected by a systematic random sampling method using the existing list of households’ from the family folder as a sampling frame of health extension works in the selected kebeles. In the case of more than one child 1–9 years old in the selected household, we select one using the lottery method and if in the selected household has no child aged 1–9 years old we used the next household.

### Data collection tools and procedures

Data was collected using a structured and pretested questionnaire from mothers or caregivers. Face to face interview was done with the mothers of children 1–9 years old children and direct observation was conducted to assess the availability and utilization of WASH facilities by trained data collectors.

Besides, trained ophthalmic nurses who participated in the 2013 global trachoma mapping project (GTMP) who are certified for trachoma grading examined the upper tarsal conjunctiva of each child by wearing 2.5x magnifying loupe and pen torch to assess each eye for the sign of active trachoma. Grading was done according to the WHO simplified grading system [3]. Eyelid eversion (turning out) of the children was done using an aseptic technique by using cotton tip applicators and alcohol used for hand disinfection. Active trachoma was defined as the presence of Trachomatous inflammation follicles (TF) or Trachomatous inflammation (TI) in either eye.

### Data quality assurance and management

The questionnaire was first prepared in English and translated to the local language Tigrigna and then it was again translated back to English. Two days Training was given for both data collectors and supervisors by principal investigator and ophthalmologist. Besides, the ophthalmic nurse has received refresher training with ophthalmologists having well experienced on the grading of trachoma using the WHO grading scheme procedures and he received practical training, PowerPoint, photographic images. One week before the actual data collection period pretest was done in 5% of the sample (25 children) from a similar population outside the study area and based on the findings of the pretest, minor modifications of questions, wordings, phrases and time required to interview respondents was made. Each questionnaire collected from the field was checked for completeness, missed values, and unlikely responses and then manually cleaned up on such indications. Then data were coded and entered into a computer using Epi-Info version 7.0.9.7 for using double-entry customizing and skip benefit, then after data cleaning, it was exported to SPSS version 20 computer software packages. Data were cross-checked for consistency and accuracy.

### Data processing and analysis

Descriptions of the main findings were done using frequencies, percentages, and summary statistics. The binary logistic regression model was fitted to assess factors associated with active trachoma. The model’s fitness was evaluated using the Hosmer -Lemeshow goodness of fit test and if the *p*-value is > 0.05 that was 0.295, and then the model was considered as fit otherwise unfit. Factors with p-value < 0.25 in chi-square (X2) cross-tabulation/bivariate analysis were entered for multivariate logistic regression analysis. Multicollinearity was checked by a variance inflation factor (VIF) for VIF > 10 to be significant Multicollinearity but in our data, no variable was with VIF more than the threshold. Backward LR stepwise regression analysis was used for variables to be independent predictors for the outcome variable. Those variables with *p*-value< 0.05 in the multivariable analysis were considered as independent predictors for active trachoma and odds ratio with a 95% confidence interval was reported.

### Operational definition

**Clean face**: a child who did not have an eye discharge or nasal discharge, fly on the face at the time of data collection [16].

**Protected water source**: water source protected by construction from outside contamination [18].

**Active trachoma**: Trachomatous inflammation follicles or Trachomatous inflammation intense [4].

**Knowledge**: mothers or caregivers of children 1–9 years old children were asked twelve knowledge related questions to assess their knowledge status on trachoma. Correct answers were given score 1 and incorrect answers 0. Mothers who score mean and above of the questions were labeled as “knowledgeable” and those who scored below the mean were labeled as “less knowledgeable” [19].

## Results

### Characteristics of participants

A total of 502 children aged 1–9 years old participated in the study making a response rate of 100%. The median age of children under study was 4 with IQR of 2–6 and more than half 266 (53%) were in the age group of 1 to 4 years old. Regarding the educational status of children, 323 (64.3%) were preschool and 172 (34.3%) were students. [Table 1].

**Table 1:** Sociodemographic characteristics of study children participated in the study conducted in Deguatemben district, Tigray, Northern Ethiopia, 2018

Variables	Variable category	Frequency (***n*** = 502)	Percentage (%)
Sex of child	Male	257	51.2
	Female	245	48.8
Education status of child	Preschool	323	64.3
	Student	172	34.3
	not attending school	7	1.4
The age group of a child	1 to 4 years old	266	53
	5 to 9 years old	236	47
Number of < 10 children in the household	≤2 children	455	90.6
	≥3 children	47	9.4

More than one-third of 190(37.8%) of mothers had no formal education. Regarding the marital and occupational status of the caregivers, 437 (87.1%) were married and 392 (78.1%) were housewives respectively. [Table 2].

**Table 2:** Socio-demographic characteristics of mothers participated in the study conducted in Deguatemben district, Tigray, Northern Ethiopia, 2018

Variables	Variable category	Frequency (***n*** = 502)	Percentage (%)
Educational Status of Mothers	No formal education	190	37.8
	1st to 4th grade	18	3.6
	5th to 8th	86	17.1
	9th to 12th	125	24.9
	college and above	83	16.5
Marital Status of mothers	Married	437	87.1
	Divorced	65	12.9
Occupational status of mothers	Housewife	392	78.1
	Government employee	34	6.8
	Merchant	76	15.1
Residency	Rural	418	83.3
	Urban	84	16.7
Age group of mothers	20-24 years	28	5.6
	25-29 years	132	26.3
	30-34 years	230	45.8
	≥35 years	112	22.3
TV/Radio in the HH	Yes	250	49.8
	No	252	50.2
Number of family in HH	≤5 families	385	76.7
	≥6 families	117	23.3
Number of under 10 children in the HH	≤2 children	455	90.6
	≥3 children	47	9.4

Four hundred forty-one (87.8%) mothers reported that they had heard about trachoma. Besides, more than half (53.2%) of mothers were knowledgeable about trachoma which is computed from the mean of correctly answered twelve questions asked.

Out of 502 households, the assessed latrine was available in 304(60.6%), and of those who have a latrine, 90% utilize it. More than 90% of the latrines are used by all members of the family. Only 61 and 36% of HHs had solid and liquid waste disposal system respectively. Forty-one percent (41%) of HHs had animals and only 56% had separate animal sheds. Regarding the source of energy for food cooking 314(62.5%) HHs use wood or animal dung and the rest use electric. More than 70% of HHs water source is from protected water source but more than 50% of HHs spent more than 30 min to fetch water. Only 94 (18.7%) of HHs wash their child’s face more than once per day. Three hundred seventy-two (74.1%) of mothers reported soap use for washing their child’s face but only 45.6% use always. [Table 3].

**Table 3:** Environmental, hygiene and sanitation characteristics of study participants in Deguatemben district, Tigray, Northern Ethiopia, 2018

Variables	Category	Frequency (***n*** = 502)*	Percentage (%)
Access to latrine	Yes	304	60.6
	No	198	39.4
Latrine utilization(*n* = 304)	Yes	295	97
	No	9	3
Who use latrine(*n* = 295)	Adults	21	7.1
	Children	4	1
	All family members	270	91.8
The solid waste disposal system	Proper pit	306	61
	Open field	196	39
Liquid waste disposal	Yes	182	36.3
	No	320	63.7
Food cooking	Kitchen	79	15.7
	Inside house	423	84.3
Window in kitchen(*n* = 79)	Yes	75	94.9
	No	4	5.1
Presence of animals in HH	Yes	207	41.6
	No	295	58.4
Animal’s living condition(*n* = 207)	Separate animal shed	117	56
	With humans	90	44
Source of energy for cooking	Wood/animal dung	314	62.5
	Electricity	188	37.5
Water source	Pipe	97	19.3
	Protected spring	267	53
	Unprotected spring	105	20.9
	River	33	6.6
Time spent to fetch water	Within compound	97	19.3
	Less than 30 min	119	23.7
	30 to 59 min	188	37.5
	More than 60 min	98	19.5
Amount of water /head/day	Less than 20 l	197	39.2
	20 to 60 l	236	47
	More than 60 l	69	13.7
Frequency of face washing	More than one per day	94	18.7
	Only once per day	384	76.5
	Not daily	24	4.8
Soap use	Yes	372	74.1
	No	130	25.9
Frequency of soap use(*n* = 372)	Always	170	45.7
	Some times	202	54.3

*n = 502 unless specified

#### Mass drug administration (MDA) related information of study participants

Out of 502 children who participated in the study 432 (86%) self-reported from mothers to have received at least one round of azithromycin during previous MDA campaigns. Out of 432 children who received azithromycin during previous MDA 359 (83%) received three rounds. **[**Table 3**].**

The prevalence of active trachoma (TF and or TI) among study participants was 21.5% (95% CI: 17.8–25.1%) in the study community. The prevalence of TF was 18.7%, TI 2.2 and 0.6% were TF and TI co-infected. The prevalence of active trachoma was slightly higher among males (52%) than among females (48%) study participants.

### Factors associated with active trachoma among 1–9 years old children

All independent variables were found statistically significant in chi-square (X2) cross-tabulation/bivariate analysis at the *p*-value of ≤0.25 considered for multivariate regression analysis **[**Table 4**].** On multivariate logistic regression, the odds of having active trachoma was found five times [AOR (95% CI) = 5.01(1.79–13.96)] higher among children 1 to 4 years old than children 5 to 9 years old. The logistic analysis also revealed that the odds of having active trachoma were almost six times [AOR (95% CI) =5.84(1.79–13.96)] higher among children who don’t use soap for face washing than their counterparts. The odds of active trachoma was more than nine times [AOR (95% CI) =9.35(4.93–67.32) higher among children who do not wash their face in daily bases compared to those who wash more than once per day. Children with unclean faces were more than eighteen times [AOR (95%CI) = 18.22(4.93–69.32)] more likely to have active trachoma than children with a clean face. The odds of having active trachoma was also 0.06 [AOR (95% CI) =0.06(0.02–0.19)] times less likely among children from mothers with good knowledge than children whose mothers were less knowledgeable. **[**Table 4**].**

**Table 4:** Factors associated with active trachoma among 1–9 years old children in Deguatemben district, Tigray, Northern Ethiopia, 2018

Factors	Active Trachoma	No Active Trachoma	COR(95%CI)	AOR(95%CI)
The age group of child				
1 to 4 years old	78	188	2.85(1.79–4.56)*	5.01(1.79–13.96)**
5 to 9 years old	30	206	1.00	
Access to latrine				
Yes	55	249	1.00	
No	53	145	1.66(1.08–2.54)*	2.21(0.82–5.98)
Solid waste disposal system				
Proper waste pit	57	249	1.00	
Open field	51	145	1.54(1.01–2.36)*	1.02(0.33–3.18)
Liquid waste disposal system				
Yes	27	155	1.00	
No	81	239	1.95(1.20–3.15)*	0.82(0.23–2.99)
Animal’s living condition				
Separate animal shed	26	91	1.00	
No separate animal shed	32	60	1.87(1.01–3.44)*	2.73(0.97–7.64)
Child face washing frequency				
More than once per day	28	66	1.00	
Only once per day	64	320	0.47(0.28–0.79)*	0.48(0.17–1.41)
Not daily	16	8	4.71(1.81–12.27)*	9.35(4.93–67.32)**
Soap use for face washing				
Yes	60	312	1.00	
No	48	82	3.04(1.94–4.78)*	5.84(1.79–13.96)**
Number of MDA rounds				
One	10	37	1.51(0.71–3.22)	1.93(0.26–14.54)
Two	10	18	3.12(1.36–7.09)*	0.40(0.02–9.21)
Three	54	302	1.00	
Clean face				
Yes	11	229	1.00	
No	97	165	12.24(6.36–23.56)*	18.22(4.93–69.32)**
Educational status of mothers				
No regular education	37	153	1.98(0.91–4.34)	0.43(0.07–2.45)
Grade 1 to 4th	4	14	2.35(0.63–8.69)	0.63(0.06–7.02)
Grade 5th to 8th	22	64	2.83(1.22–6.58)*	0.53(0.07–3.88)
Grade 9th to 12th	36	89	3.33(1.51–7.35)*	1.91(0.28–13.28)
College and above	9	74	1.00	
Mothers knowledge on trachoma				
Knowledgeable	26	241	0.20(0.12–0.33)*	0.06(0.02–0.19)**
Less knowledgeable	82	153	1.00	

* = significant on bivariate, ** = significant on multivariable analysis at *p*-value < 0.05

*COR* crude odds ratio and *AOR* adjusted odds ratio, 1.00 = referent.

## Discussion

The prevalence of active trachoma in the district was found to be 21.5% (17.8–25.1%) 1 year after three rounds of mass drug administration. Though there is a decline in the prevalence of TF from 44.7% in the baseline to 18.7% after three rounds of MDA, it is far above the 10% treatment threshold [11]. This might be related to the effectiveness of the trachoma control program of MDA with a high coverage rate.

The prevalence of active trachoma in our study area was consistent with finding from district level studies in different parts of the country including; Baso Liben 24.1%, Gongi Kollela 23.1%, Demba 18%, Kersa 25.2%, Dera 18.6%, Cheha districts 22.8% and Maksegnit Town 23.8% [15, 16, 19–23]. But the prevalence in our study area was lower than the district level study conducted in Ankober 53.9%, Gazegibela 52.4%, Zala 36.7% [12, 13, 24]. This might be the provision of Mass drug administration in our study area was better effective compare to those study areas.

In contrast, the prevalence of active trachoma among children in our study is higher than the study findings of Ghana 5.6 and 3.5%, and Gambia 2.8% after control activities [25, 26]. Besides, it is higher than the study findings of evaluation units of Welkait, Tsegedae, Tahtay, and Laelay-Adiabo even without any intervention according to the GTMP result of Tigray region [11]. This difference could be attributed due to the difference in the study setting, study period, intervention and baseline difference in the prevalence of active trachoma in the communities. In addition to this, hyper-endemic districts may require more rounds of MDA with an even high coverage rate as suggested by West SK. et al.,2011 [27].

Active trachoma was five times more likely to happen among children from 1 to 4 years old than children 5 to 9. This finding is in line with the findings of the study conducted in Ankober and southern nations and nationalities people (SNNP) [24, 28]. This may be due to young children may not able to care about themselves and play in dirty places than older children.

Children who do not use soap for face washing were almost six times to have active trachoma than their counterparts. This finding was in line with the study finding conducted in Baso Liben district [19]. This might be due to the ability of soap to clear microorganisms.

Children who do not wash their faces on a daily bases were found to be more than nine times to have active trachoma compared to those who wash their faces more than once per day. This result is consistent with the study findings of Baso Liben, Gonji kollela, Gazigeabella, and Zala districts [12, 13, 19, 21]. This might be due to the habit of face washing may affect the transmission of the disease from person to person.

The finding of this study also revealed that children with unclean faces were more than eighteen times to have active trachoma than their counterparts. Other studies also reported similar findings which include; a study conducted in Dembia and Cheha districts [15, 16]. This may be due to the ability of the secretions to attract eye-seeking flies which increases the risk of transmission trachoma from one person to another [29–31].

Children from knowledgeable mothers were found to be 0.07 times less likely to have active trachoma compared to children from less knowledgeable mothers. This finding was in line with the study result from Baso Liben and Zala districts [13, 19]. The possible explanation for this difference could be the difference in access to information, education and communication media on trachoma prevention, community-based health education by trained health workers.

### Strength and limitations of the study

As the strength of our study, Tetracycline eye ointment was provided to those who had active trachoma two tubes to apply for 6 weeks. Being community-based cross-sectional and the grading of trachoma was done by certified, having more experience in trachoma grading and participated in the pre-intervention assessment of the global trachoma mapping project in the Tigray region. However, the study might have some limitations that first; the cross-sectional nature of the study design does not confirm the definitive cause and effect relationship. For those children who are older, we have not taken into consideration their practice on face washing. The questionnaires were designed and translated into a local language and re-translated to English. This could be also a recipe for bias. Furthermore, the study may prone to reporting bias since some of the data are collected based on self-reported information.

## Conclusion

Although the prevalence of active trachoma among 1–9 years old children indicate a decline 1 year after three rounds of MDA in the district, it is still higher than the WHO threshold prevalence of 20% which is used to determine trachoma as a severe public health problem and it is far from the elimination of trachoma as a public health problem in a community as when there is less than 5% active trachoma in children [32, 33]. Not washing the face daily, not using soap for face washing; unclean face, younger child age and mother’s knowledge on trachoma were found to be independent predictors of active trachoma in the district 1 year after 3 years of MDA with azithromycin.

The regional health bureau and woreda health office should implement all components of the SAFE strategy emphasizing personal hygiene (facial cleanness) and environmental improvement.

## Data Availability

All the data supporting the findings is contained within the manuscript, when there is in need the data-set used for the present study’s conclusion can be accessible from the corresponding author on reasonable request.

## Abbreviations

TF: Trachomatous inflammation Follicular
TI: Trachomatous inflammation intense
TS: Trachomatous scarring
TT: Trachomatous Trichiasis
CO: Corneal opacity
SAFE: Surgery, antibiotics, facial cleanness, and environmental improvements
WHO: World health organization; HH: household
